# Neurologic effects of exogenous saccharides: A review of controlled human, animal, and *in vitro* studies

**DOI:** 10.1179/1476830512Y.0000000004

**Published:** 2012-07

**Authors:** Erika D. Nelson, Jane E. Ramberg, Talitha Best, Robert A. Sinnott

**Affiliations:** 1 Mannatech, Incorporated, Coppell, TX, USA; 2 Nutritional Physiology Research Centre, Sansom Institute for Health Research, University of South Australia, Adelaide, Australia; 3 Centre for Human Psychopharmacology, Swinburne University of Technology, Victoria, Australia

**Keywords:** Saccharide, Memory, Synaptic plasticity, Prebiotic, Neurodegeneration, Glucan, Fucoidan

## Abstract

**Objectives:**

Current research efforts are centered on delineating the novel health benefits of naturally derived saccharides, including growing interest in their abilities to influence neurologic health. We performed a comprehensive review of the literature to consolidate all controlled studies assessing various roles of exogenous saccharide compounds and polysaccharide-rich extracts from plants, fungi, and other natural sources on brain function, with a significant focus on benefits derived from oral intake.

**Methods:**

Studies were identified by conducting electronic searches on PubMed and Google Scholar. Reference lists of articles were also reviewed for additional relevant studies. Only articles published in English were included in this review.

**Results:**

Six randomized, double-blind, placebo-controlled clinical studies were identified in which consumption of a blend of plant-derived polysaccharides showed positive effects on cognitive function and mood in healthy adults. A separate controlled clinical study observed improvements in well-being with ingestion of a yeast beta-glucan. Numerous animal and *in vitro* studies have demonstrated the ability of individual saccharide compounds and polysaccharide-rich extracts to modify behavior, enhance synaptic plasticity, and provide neuroprotective effects.

**Discussion:**

Although the mechanisms by which exogenous saccharides can influence brain function are not well understood at this time, the literature suggests that certain naturally occurring compounds and polysaccharide-rich extracts show promise, when taken orally, in supporting neurologic health and function. Additional well-controlled clinical studies on larger populations are necessary, however, before specific recommendations can be made.

## Introduction

The increasing prevalence of debilitating neurodevelopmental, psychiatric, and neurodegenerative diseases continues to be of significant concern for the developed world.^1^ Owing to the paucity of affordable, safe, and effective treatment options for neurologic disorders, there is considerable interest in preventative and therapeutic complementary and alternative medicines. According to a recent survey, 21% of American adults with common neurologic conditions (memory loss, back pain with sciatica, migraines, regular headaches, seizures, stroke, and dementia) use biologically based therapies, consisting primarily of herbal remedies.^2^ Traditional Chinese, Ayurvedic, and African medicines have utilized an assortment of plants and herbal extracts to treat various central nervous system (CNS) dysfunctions.^3^ Effective compounds from well-known neurobiologically active plants include flavonoids from *Gingko biloba* and *Hypericum perforatum* (St John's wort) as well as alkaloids, quinones, tannins, and other phenolic compounds. To date, very little attention has been given, however, to exploring the efficacy of naturally derived complex carbohydrate compounds. (For the sake of this review, the term complex carbohydrates will be used interchangeably with the terms glycans, polysaccharides, fiber, and complex sugars or saccharides.)

Saccharide molecules play a number of fundamentally important roles in the brain, where they are largely glycoconjugated to proteins or lipids to assist in structural development, synaptogenesis, and synaptic transmission.^4^ Saccharides can also be used as a source for energy and neurotransmitter production.^5^ An early review highlighted the potential role and impact of saccharides in both simple and complex glycoconjugated form for supporting brain and cognitive function.^6^ Indeed, exogenously applied small sugar molecules, specifically monosaccharides, have been shown to impact functions within the brain related to cognitive benefits in human and animal studies. For example, consumption of glucose, a monosaccharide with well-established effects on brain function, has significant impacts on mood and cognition in human subjects.^7^ Systemic and intracranial application of l-fucose have been shown to influence glycoprotein synthesis and enhance memory behavior and synaptic plasticity in rodents,^8,9^ whereas sialic acid, another monosaccharide component of glycoproteins, has been shown to improve learning and memory following oral administration to piglets.^10^ Given the high concentrations of sialic acid in both the brain and human breast milk, it has been hypothesized that this monosaccharide may be an important nutrient for infant brain development.^11^

The exogenous application of more complex sugar compounds has also been studied with respect to impacts on the brain. However, there remains a limited understanding of the potential mechanisms by which complex saccharide molecules may be influencing neurologic function. Saccharides derived from natural sources are structurally heterogeneous, come in neutral and acidic varieties, and can contain as much as 10 different monosaccharide sugars. The diversity of saccharides in both form and source provides potential for applied research into the occurrence, structure, and health properties of saccharides. In particular, varieties of saccharide compounds have been shown to improve brain function *in vitro* and in human and animal studies following oral, systemic, and localized administration. To better understand the efficacy and possible mechanisms of action by which complex saccharides influence CNS function, we thus conducted this review of the available literature. It is anticipated that this information may be useful for guiding future research that will eventually provide a clear understanding of safe and effective dietary complex saccharides for specific neurologic health applications.

## Dietary polysaccharides improve cognitive function and mood in healthy young and middle-aged adults

A number of preliminary randomized, double-blind, placebo-controlled clinical trials have indicated that consumption of naturally sourced polysaccharides can benefit brain function in healthy young and middle-aged adults (Table 1). Supplementation with a baker's yeast beta-glucan product (Wellmune WGP^®^; Biothera, Eagan, MN, USA) for 4 weeks improved various measures of well-being in healthy adult marathon runners compared with placebo.^12^ Specifically, profile of mood state scores of tension, fatigue, anger, and confusion were significantly decreased and scores of vigor were significantly increased following supplementation. Significant improvements in cognitive function and mood have also been found in healthy middle-aged adults with a mixed polysaccharide product (Ambrotose^®^ complex; Mannatech, Incorporated, Coppell, TX, USA). In a recent study, individuals supplemented their diets for 12 weeks with placebo or a blend of saccharide-rich extracts from plants including *Aloe vera, Astragalus gummifer*, and *Anogeissus latifolia*. When compared with placebo, those subjects who consumed the blend of plant polysaccharides performed significantly better on tasks of recall and recognition memory and reported significantly reduced scores for tension and low mood (depression-dejection scale).^13^

**Table 1:** Saccharide consumption improves cognitive function and mood in healthy adults

Polysaccharide(s) (product name)	Source(s)	Study design	Population	*N*	Dose	Duration	Cognitive tests performed	Significant effects	Reference
Beta-1,3/1,6 glucan (Wellmune WGP^®^)	*Saccharomyces cerevisiae*	Randomized, double-blind, placebo-controlled	Healthy adult marathon runners	75	250 or 500 mg/day	4 weeks	POMS	250 mg reduced tension and fatigue scores at 4 weeks and reduced confusion scores at 2 and 4 weeks; 500 mg reduced anger scores at 2 weeks, reduced fatigue, tension and confusion scores at 2 and 4 weeks and increased vigor scores at 2 and 4 weeks	12
Mixed polysaccharide product (Ambrotose^®^ complex)	*Aloe barbadensis, Larix* sp.*, Anogeissus latifolia, Astragalus gummifer, Oryza sativa*, glucosamine HCl	Randomized, double-blind, placebo-controlled	Healthy, middle-aged adults	109	3.6 g/day	12 weeks	RAVLT; Visual Pattern Span Recall; Visual Pattern Span Recognition Reading Span; Computation Span; Stroop; Letter Cancellation; Digit Symbol Coding, Boxes test, Matrix Reasoning & Spot the Word (Weschler Adult Intelligence Scale III); POMS; Depression Anxiety and Stress Scale; Perceived Stress Scale-10	Better performance on immediate recall tasks [RAVLT trials 2 and 5] and recognition memory task [RAVLT Recognition]; lower depression-dejection and anger-hostility scores [POMS]	13
				73	4 g	2 hours	RAVLT; Cognitive Demand battery (Serial Threes; Serial Sevens, Rapid Visual Information Processing, Visual analog mental fatigue scale); POMS; Short-form health survey (SF-36); State-trait anxiety questionnaire; Bond–Lader visual analog scale	Better performance on recognition memory task [RAVLT recognition A & B] and working memory task [Serial Sevens] during mental fatigue. Effects were independent of blood glucose response	14
				45	7 g	10 minutes	RAVLT; Self-Ordered Pointing; Digit Span forwards; Digit Span backwards; Matrix Reasoning (Weschler Adult Intelligence Scale III)	No effects. 25 g glucose also showed no effects	15
		Randomized, double-blind, placebo-controlled, crossover	Healthy college students	30	1 tablespoon (approximately 4 g)	45 minutes	Same-Different visual discrimination; Standard Progressive Matrices; Stroop	Better performance on visual discrimination task (Same–Different)	16
				32	1 tablespoon (approximately 4 g)	45 minutes	Reading span; operation span	Better performance on simple working memory task (reading span, first session)	16
			Healthy male college students	20	1 tablespoon (approximately 4 g)	30 minutes	EEG recordings during focus on a stationary visual target	Enhanced power in theta, alpha, and beta brain wave frequencies associated with attention and arousal	17

EEG, electroencephalogram; POMS, Profile of Mood States questionnaire; RAVLT, Rey Auditory-Verbal Learning Test.

Interestingly, acute effects on brain function have also been observed following intake of plant polysaccharides. A single dose of Ambrotose^®^ complex administered to healthy, middle-aged adults has been recently shown to improve recognition memory and working memory despite conditions of mental fatigue.^14^ In this study, blood glucose-mediated effects on cognitive function were ruled out since no changes in blood glucose levels were observed before or during testing, at 30 and 105 minutes post-treatment, respectively. Earlier research in middle-aged adults showed that a single dose of Ambrotose^®^ complex had a tendency towards positive effects on memory tasks; however, these differences were not statistically significant, which may have been due to methodological differences in dose and time between supplementation and testing (10 minutes).^15^ Two studies of young adults demonstrated improved performance on tasks of visual discrimination and working memory at 45 minutes following intake of Ambrotose^®^ complex.^16^ The exact mechanisms of action behind these acute improvements in cognitive function and mood are unclear. However, in a separate study of young male adults, significant increases in electroencephalogram-recorded brain wave frequencies during focused attention were seen 30 minutes after consumption of Ambrotose^®^ complex relative to placebo.^17^ The specific changes in brain wave frequencies observed are commonly associated with abilities such as concentration and arousal. Overall, supplementation with a blend of plant polysaccharides seems to benefit various aspects of neurologic function and suggests functional benefits in healthy young and middle-aged adults. It is important to note, however, that these clinical studies, although well-controlled, were performed on relatively small populations; larger study groups are needed to confirm the validity of these results.

## Exogenous saccharides modulate memory- and mood-related behaviors in rodents

Given the mechanistic limitations associated with clinical research, rodent models are commonly utilized to study the effects of therapeutic interventions on the CNS. A variety of rodent behavioral tests can be significant indicators of human cognitive function, which, when combined with experimentation on tissues from specific brain regions, can help elucidate possible mechanisms of action. For studies looking at behavioral and neurobiological effects of natural saccharides, animals with neurologic impairments have been utilized, which serves to both study the efficacy for protection against impairment-induced functional deficits and increase the likelihood of observing positive results (Table 2). For example, oral administration of isolichenan, an alpha-glucan from the lichen *Cetrariella islandica*, to ethanol-fed mice was able to reverse the ethanol-induced impairment in memory acquisition assessed by passive avoidance tests.^18^ Furthermore, an attenuation of spatial memory deficits during testing in the Morris water maze were observed with isolichenan ingestion in rats exposed to beta-amyloid peptide (A-beta), a small protein associated with the neurologic deterioration of Alzheimer's disease. It is important to note that in both studies, naive animals – those unexposed to either ethanol or A-beta – did not display memory enhancements with isolichenan treatment.

**Table 2:** Saccharides modify rodent behavior

Polysaccharide(s)	Source(s)	Animals	Dose/day	Duration	CNS tests performed	Significant effects	Reference
Alpha-glucan (isolichenan)	*Cetrariella islandica*	5-week-old ♂ Std-ddY mice treated with 30% ethanol p.o. 20 minutes before learning trial	100, 200, or 400 mg/kg p.o. once	30 minutes before learning trial	Passive avoidance step through and step down	All doses reversed ethanol impaired fear memory acquisition; no effects on mice without ethanol exposure	18
		♂ Wistar rats receiving daily i.c.v. injections of A-beta peptide from day 7 to day 4	100 or 200 mg/kg p.o.	8 days	Morris water maze	100 mg/kg reversed A-beta-induced spatial memory deficits; no effects on rats without A-beta peptide pretreatment	18
Arabinoxylan; beta-glucan	*Triticum aestivum* L.; *Hordeum vulgare* L.	♂ Sprague–Dawley rats with bilateral common carotid artery occlusion	20 mg/kg p.o.	Days 8–14 post-surgery; testing on days 22–26 post-surgery	Morris water maze; immunohistochemistry and western blot (GFAP, MBP); Luxol fast blue and hematoxylin staining	Both treatments reversed ischemia-induced spatial memory deficits; arabinoxylan prevented loss of MBP in corpus callosum	20
Pectin	Citrus [peel]	9–11-week-old C56BL/6J mice injected i.p. with 100 µg/kg LPS	10% pectin diet	6–8 weeks post-weaning	Social exploratory behavior of a novel juvenile mouse	Faster recovery from LPS-induced social withdrawal compared with 5% cellulose diet	19
Polysaccharide fraction: PSF (no characterization provided)	*Panax ginseng*	Adult ♂ Wistar rats	4, 20, or 40 mg/kg i.p.	10 days	Conditioned Active Escape Response	All doses differentially improved indications of learning and memory	22
Alpha-glucan (FPS)	Fu Zi (*Aconitum carmichaeli* Debx)	8–11-week-old ♂ C57BL/6 mice	5 to 400 mg/kg i.p.	Varied with assay: 30 minutes, 6 hours, or 1–4 weeks	BrdU labeling; forced swim; open-field; novelty-suppressed feeding; chronic social defeat stress; monoamine levels in the frontal cortex; ELISA and Western blot (BDNF)	10–400 mg/kg doses increased newborn neurons in the DG; 50 and 100 mg/kg produced AD effects (similar to imipramine); 100 mg/kg increased BDNF expression in the hippocampus; AD and neurogenesis effects were blocked by co-injection with K252a (trkB inhibitor)	23

AD, antidepressant; BrdU, 5-bromo-2′-deoxyuridine; DG, dentate gyrus; ELISA, enzyme-linked immunosorbent assay; GFAP, glial fibrillary acidic protein; i.c.v., intracerebroventricular injection; i.p., intraperitoneal injection; MBP, myelin basic protein; p.o., oral administration.

A recent study looking at the effects of dietary fiber on sickness behavior demonstrated faster recovery from lipopolysaccharide (LPS)-induced social withdrawal with a pectin diet compared with a cellulose diet.^19^ Although cellulose also falls within the complex saccharide category, this study was concerned with comparing the immunological benefits of soluble (pectin) versus insoluble (cellulose) dietary fiber. Their results suggest that effects within the gastrointestinal tract following consumption of soluble, fermentable carbohydrates may be important for the neuroimmune recovery from LPS. Gastrointestinal fermentation effects were also proposed as one possible mechanism by which orally administered arabinoxylan from the yeast *Triticum aestivum* and beta-glucan from barley were able to preserve memory in a mouse model of vascular dementia.^20^

Neurologic effects of polysaccharide-rich plant extracts have also been observed in rodents following systemic injection (Table 2). The biological activity of Asian ginseng (*Panax ginseng*), a traditional Chinese medicine and popular natural product with touted cognitive health benefits, is typically attributed to its ginsenoside saponin content.^21^ However, some research suggests that ginseng's saccharide components may also play a role. A polysaccharide fraction of *P. ginseng* administered by daily intraperitoneal injections for 10 days seemed to enhance learning in healthy adult rats.^22^ Possible mechanisms of action were not addressed by the authors of this study. Fu Zi, a preparation of the daughter root from the plant *Aconitum carmichaeli* Debeaux (Chinese Monkshood), is another traditional Chinese medicine that has been used for centuries to treat mood disorders. Intraperitoneal injections of an alpha-glucan isolated from Fu Zi (FPS) showed antidepressant-like effects in the forced swim test, similar to the tricyclic antidepressant imipramine, in healthy mice.^23^ Surprisingly, FPS was also able to reverse the social avoidance behavior of defeated mice, a model of chronic stress, within just 2 weeks of intraperitoneal administration, whereas the effects of imipramine treatment were only observed after 4 weeks. The authors attributed these behavioral effects to increased brain-derived neurotrophic factor (BDNF) signaling and neurogenesis within the hippocampus, both known to be associated with an antidepressant-like response.^24^

## Alpha- and beta-glucans enhance hippocampal synaptic plasticity

The hippocampus is an important brain structure that helps regulate many behaviors, including memory and mood, and contains one of only a few sites of adult neurogenesis, the dentate gyrus. Owing to its organized neuronal circuitry, the hippocampus is commonly utilized to measure various types of synaptic transmission, including a form of synaptic plasticity – long-term potentiation (LTP) – that is believed to be the cellular basis for learning and memory. LTP is observed as an activity-dependent increase in synaptic strength that takes place when repeated presynaptic neurotransmitter release immediately precedes the generation of an action potential by the postsynaptic neuron, rendering the postsynaptic neuron hypersensitive to subsequent stimulation. It is thought that this repeated stimulation represents learning during exposure to a novel stimulus, which then strengthens specific synaptic connections for encoding long-term memories. Some of the first *in vivo* electrophysiological recordings of LTP, performed on anesthetized rabbits, were of the responses of hippocampal dentate gyrus neurons receiving synaptic inputs from the entorhinal cortex.^25^

Utilizing a similar method of recording hippocampal LTP in rats, a number of studies have found significant effects of lichen- and fungal-derived glucans on synaptic plasticity (Table 3). Acute exposure (≤30 minutes) to the alpha-glucans PC-2 and PB-2 from the lichens *Parmelia caperata* and *Flavoparmelia baltimorensis*, respectively, enhanced the magnitude of LTP induction when administered either orally or intravenously.^26,27^ Intravenous administration of another lichen-derived alpha-glucan, isolichenan from *Cetariella islandica*, also enhanced synaptic plasticity in the dentate gyrus.^18^ In this case, these results correlated somewhat with isolichenan's ability to reduce memory impairments following oral administration (see Table 2). Interestingly, although the effects of PB-2 and PC-2 on synaptic plasticity were seen with both oral and intravenous administration, no changes were seen when the alpha-glucans were applied directly to the brain via intracranial ventricular injection. Owing to these results, the authors proposed a peripheral site of action for alpha-glucans, which then leads to unidentified centralized signaling mechanism(s) that can elicit the observed effects on synaptic activity. Additional studies suggest an involvement of norepinephrine, both centrally and peripherally. Co-intravenous injection or pretreatment with specific beta-1-adrenergic receptor antagonists, by either intracranial ventricular injection or direct infusion into the dentate gyrus, resulted in inhibition of PB-2's ability to enhance LTP.^28,29^ These findings are further supported by the observation that PC-2 had no effects on synaptic plasticity in adrenalectomized rats.^26^ Activation of brain interleukin-1 receptors (IL-1R) may also play a role, since intracranial ventricular injection of IL-1R antagonist prior to intravenous PB-2 application was shown to enhance the alpha-glucan's effects on LTP.^28^

**Table 3:** Glucans enhance synaptic plasticity in the dentate gyrus of the rat hippocampus

Polysaccharide(s)	Source(s)	Animals	Dose	LTP protocol	Significant effects	Reference
Alpha-glucan (PC-2)	*Parmelia caperata*	Anesthetized 7–8-week-old ♂ Wistar rats	125 or 250 mg/kg p.o.	30 minutes before weak tetanic stimulation (20 pulses at 60 Hz)	250 mg/kg enhanced the magnitude of LTP for up to 40 minutes	26
		Anesthetized 7–8-week-old ♂ normal and adrenolectomized Wistar rats	0.1–5 mg/kg i.v.	15 minutes before weak tetanic stimulation (20 pulses at 60 Hz)	1 mg/kg enhanced the magnitude of LTP induction in normal but not adrenolectomized rats	26
		Anesthetized 7–8-week-old ♂ normal Wistar rats	10–1000 ng i.c.v.	Not provided	LTP unchanged (data not shown)	26
Alpha-glucan (PB-2)	*Flavoparmelia baltimorensis*	Anesthetized 5–6-week-old ♂ Wistar/ST rats	50, 100, or 200 mg/kg p.o.	20 minutes before weak tetanic stimulation (30 pulses at 60 Hz)	100 and 200 mg/kg enhanced the magnitude of LTP induction	27
			0.1–10 mg/kg i.v.	20 minutes before weak tetanic stimulation (30 pulses at 60 Hz)	1 and 5 mg/kg enhanced the magnitude of LTP induction	27
			1 or 2 µg/brain i.c.v.	20 minutes before weak tetanic stimulation (30 pulses at 60 Hz)	LTP unchanged	27
		Anesthetized 6–9-week-old ♂ Wistar rats	5.0 mg/kg i.v.	15 minutes before weak tetanic stimulation (30 pulses at 60 Hz)	Enhanced the magnitude of LTP induction. This effect was blocked by i.v., i.c.v. or DG injections of atenolol (adrenergic beta-1 receptor antagonist)	29
		Anesthetized 5–8-week-old ♂ Wistar rats	1.0 mg/kg i.v.	15 minutes before weak tetanic stimulation (30 pulses at 60 Hz)	Enhanced the magnitude of LTP induction. This effect was further enhanced by i.v. or i.c.v. injections of IL-1Ra and inhibited by i.c.v. metropolol (adrenergic beta-1 receptor antagonist)	28
Alpha-glucan (isolichenan)	*Cetrariella islandica*	Anesthetized ♂ Wistar rats	1 mg/kg i.v.	15 minutes before weak tetanic stimulation (20 pulses at 60 Hz)	Enhanced the magnitude of LTP induction	18
Beta-1,3/1,6 glucan (lentinan)	*Lentinula edodes*	Anesthetized 7–8-week-old ♂ Wistar rats	200 mg/kg p.o.	30 minutes before weak tetanic stimulation (20 pulses at 60 Hz)	Enhanced the magnitude of LTP induction	30
			1 mg/kg i.v.	15 minutes before weak tetanic stimulation (20 pulses at 60 Hz)	Enhanced the magnitude of LTP induction	30

DG, dentate gyrus; i.c.v., intracerebroventricular injection; i.v., intravenous injection; p.o., oral administration.

Like alpha-glucans, beta-glucans also appear to modulate synaptic plasticity (Table 3). Oral and intravenous administration of lentinan, a beta-1,3/1,6 glucan from the fungus *Lentinula edodes*, and oral administration of Hoelen, a beta-glucan from the fungus *Poria cocos* Wolf, resulted in increased magnitudes of LTP.^30,31^ Altogether, there is a fair amount of *in vivo* evidence suggesting alpha- and beta-glucan consumption can lead to positive effects on synaptic activity in the dentate gyrus of the hippocampus. Additional research is needed to determine whether these saccharide-induced changes in synaptic plasticity convincingly lead to cognitive outcomes, and whether these functional and behavioral effects are mediated by norepinephrine or IL-1 receptor activation in the hippocampus.

## Neuroprotective effects of plant-derived saccharides

Neurodegenerative diseases, including Alzheimer's, Parkinson's, and Huntington's, are devastating disorders that result from progressive loss of neurons in various brain regions that control motor and cognitive function. Aging is the greatest risk factor for these diseases. Identifying treatments to prevent neuronal loss has proven a daunting task, and as the average life expectancy of the human population continues to increase, the need for successful therapies intensifies. An encouraging number of animal and *in vitro* studies have demonstrated neuroprotective effects of plant-derived saccharides and saccharide-rich extracts (Table 4).

**Table 4:** Saccharides and saccharide-rich extracts exert neuroprotective effects *in vitro* and *in vivo*

Polysaccharide(s)	Source(s)	Neuronal preparation	Dose	Duration	Tests performed	Significant effects	Reference
Trehalose	*Saccharomyces cerevisiae*	Cultured mouse neuroblastoma Neuro2a cells with tNhtt-eGFP expression (cellular model of Huntington's disease)	50 µM	3 days	tNhtt aggregation; MTT assay	Decreased tNhtt aggregation & increased cell viability	32
		3-week-old heterozygous huntington exon-1-transgenic mice, strain R6/2 (bearing 145 CAG repeats)	2% in drinking water	12 weeks	Nissl staining and immunohistochemistry (ubiquitin); rotarod; foot-printing; paw clasping phenotype	Decreased striatal atrophy and intranuclear polyglutamine aggregates in the motor cortex and striatum; improved motor dysfunction, paw clasping phenotype and survival	32
		3-week-old heterozygous huntington exon-1-transgenic mice, strain R6/2 (bearing 145 CAG repeats) injected i.c.v. with murine neural progenitor cell line C17.2	2% in drinking water	10 weeks (cells injected at week 8)	Immunocytochemistry (Nestin, MAP-2, ubiquitin); western blot (expanded polyQ); paw-clasping phenotype; foot printing; rotarod; Y-maze	Trehalose alone and in combination with C17.2 cells decreased polyglutamine aggregates in striatum, increased striatal volume, extended life span, delayed onset and severity of paw clasping, improved motor function; combination treatment was more effective and also improved memory performance	33
		3-month-old ♂ or 14-month-old ♀ PK− / −/TauVLW mice (model of tauopathy with Parkinsonism)	1% in drinking water	3–12 weeks	Actimeter; stride length; Y-maze; immunohistochemistry (TH, total and phosphorylated tau, GFAP, A-beta); western blot; monoamine levels; glutathione assay	Trehalose improved motor function and anxiety and ameliorated tau pathology and astrogliosis; reversed dopaminergic deficits at 10 weeks; autophagy markers were increased	34
Fucoidan	*Laminaria japonica*	Cultured mouse dopaminergic cell line, MN9D, exposed to the neurotoxin MPP+	0.01, 0.1, or 1.0 mg/ml	1 hours pretreatment + 36 hours cotreatment with MPP+	LDH activity; MTT assay; morphology	0.1 and 1.0 mg/ml doses reversed neuronal injury caused by MPP +	35
		8–10-week-old ♂ C57BL/6 mice injected i.p. with MPTP (model of Parkinson's)	12.5 or 25 mg/kg i.p.	18 days (MPTP injection on day 11)	Locomotor activity; dopamine, DOPAC and HVA levels in the striatum; immunohistochemistry and western blot (TH); oxidative stress and antioxidant capacity in the substantia nigra	Both doses differentially rescued locomotor deficits, prevented striatal depletion of dopamine and DOPAC, protected against the loss of TH-positive neurons and lipid peroxidation, and enhanced antioxidant activities in the substantia nigra	35
	*Fucus vesiculosus*	Acutely dissociated Sprague–Dawley rat diagonal band of Broca forebrain neurons exposed to A-beta; primary rat basal forebrain cultures exposed to A-beta	Ranging from 50 to 1 µM	Active perfusion; 24 hours pretreatment or 48 hours cotreatment with A-beta	Whole-cell patch clamp; MTT and live/dead assays; immunohistochemistry (VAchT); electron microscopy for A-beta aggregation; DCF fluorescence; western blot (cleaved caspase-3; PKC phosphorylation)	1 µM blocked the A-beta-induced reduction in whole-cell currents; 0.1 and 1 µM differentially protected against A-beta-induced apoptosis; 10 µM-induced apoptosis	36
		7-day-old rats with unilateral cerebral hypoxia-ischemia	25–500 mg/kg i.p. twice	Immediately before and after hypoxia exposure	MPO activity; RT-PCR and western blot (P-selectin); Nissl stain of brain slices	25, 50, and 100 mg/kg doses attenuated brain damage induced by hypoxia-ischemia; >100 mg/kg doses exhibited toxic side effects	38
		♂ Sprague–Dawley rats with bolus collagenase-induced ICH	30 µg/hour i.v. infusion	7 days, beginning 30 minutes after ICH	MRI; spontaneous circling, ability to traverse a wooden beam; forelimb postural reflex; independent forelimb reaching and grasping in a staircase apparatus; passive avoidance	Increased size of hematoma, but improved motor function and memory retention	37
Polysaccharide fractions: J2 (xyloglucan); J3 (1.0 Rha: 1.3 Ara: 4.1 Xyl: 3.6 Gal: 9.1 Glu); J4 (0.3 Rha: 0.8 Ara: 0.1 Xyl: 0.1 Gal)	*Nerium indicum*	Primary rat cortical cultures exposed to serum-free media or A-beta peptides	20 or 40 µg/ml of J2, J3, or J4	24 hours with serum-free media; 1 hours pretreatment + 24 hours with A-beta	DAPI staining; caspase-3 activity; western blot (PDK-1 and Akt phosphorylation)	All fractions and doses protected against serum-deprivation-induced and A-beta-induced apoptosis	39
Polysaccharide fraction: J6 (1.2 Rha: 2.0 Ara: 1.0 Xyl: 2.0 Gal)		Primary rat cortical cultures exposed to A-beta peptides	Ranging from 20 to 500 µg/ml	1 hour pretreatment + 24 hours cotreatment with A-beta	DAPI staining; caspase-3 activity; LDH activity; western blot (cleaved caspase-3; p38 MAPK and Akt; JNK, MAPK and Akt phosphorylation)	100, 250, or 500 µg/ml doses protected against A-beta-induced apoptosis; reduced cytotoxicity	40
Polysaccharide fraction: LBA (6.2% w/w protein, 61 neutral sugars; 35.1 M% Ara, 10.0 Rha, 4.0 Xyl, 1.3 GluAc, 23.9 GalAc, 0.8 Man, 16.0 Gal, 8.9 Glu)	*Lycium barbarum*	Primary rat cortical cultures exposed to A-beta peptides	Ranging from 0.0001 to 100 µg/ml	1 hour pretreatment + 24 hours cotreatment with A-beta; 1 hour pretreatment only (washout)	DAPI staining; morphology; caspase-3 activity; LDH activity; western blot (cleaved caspase-3; JNK and C-Jun phosphorylation)	0.1, 1, 10, or 100 µg/ml differentially protected against A-beta-induced apoptosis	42
		Primary rat cortical cultures exposed to glutamate	Ranging from 0.1 to 500 µg/ml	24 hours with glutamate; 1 hour glutamate + 23 hours post-treatment	LDH activity; trypan blue stain; caspase-3 activity; western blot (JNK phosphorylation); nitroblue tetrazolium reduction	10, 100, 250, or 500 µg/ml co- and post-treatment differentially protected against glutamate-induced cell death	44
		Primary rat cortical cultures exposed to Hcy	Ranging from 0.1 to 500 µg/ml	1 hour pretreatment + 24 hours Hcy; 1, 2, or 4 hours Hcy + 23, 22, or 20 hours post-treatment	DAPI staining; caspase-3 activity; LDH activity; western blot (cleaved caspase-3 and tau; tau, JNK, GSK-3 beta and ERK phosphorylation)	100, 250, or 500 µg/ml pre- and post-treatment differentially protected against homocysteine-induced cell death	45
Polysaccharide fractions: LBB (4.99%w/w protein, 22 neutral sugars; 32.1 M% Ara, 31.2 Xyl, 1.4 GluAc, 3.2 GalAc, 2.9 Man, 15.9 Gal, 13.3 Glu); LBB-1 (3.35%w/w protein, 84 neutral sugars; 20.2 M% Ara, 8.1 Rha, 32.9 Xyl, 8.0 GluAc, 8.2 GalAc, 1.0 Man, 15.6 Gal, 5.3 Glu); LBB-II (5.82%w/w protein, 75 neutral sugars; 14.1 M% Ara, 15.8 Rha, 29.1 Xyl, 3.5 GluAc, 22.6 GalAc, 1.0 Man, 8.4 Gal, 5.5 Glu)		Primary rat cortical cultures exposed to A-beta peptides	Ranging from 0.1 to 500 µg/ml	1 hour pretreatment + 24 hours cotreatment with A-beta	DAPI staining; caspase-3 activity; LDH activity; western blot (cleaved caspase-3; JNK and Akt phosphorylation)	10, 100, or 500 µg/ml LBB, 10 or 100 µg/ml LBB-I or LBB-II differentially protected against A-beta-induced apoptosis	41
Polysaccharide fractions: LBP (8.6%w/w protein, 18 neutral sugars; 16.1 M% Ara, 4.3 Rha, 2.8 Xyl, 14.4 GalAc, 1.5 Man, 12.9 Gal, 47.7 Glu, 0.3 NAcGlu); LBP-III (6.3%w/w protein, 15 neutral sugars; 6.1 M% Ara, 1.5 Rha, 92.4 GalAc)		Primary rat cortical cultures exposed to A-beta peptides	Ranging from 10 to 500 µg/ml	1 hour pretreatment + 24 hours cotreatment with A-beta	Caspase activity; western blot (PKR phosphorylation)	100 and 500 µg/ml LBP or LBP-III protected against A-beta-induced apoptosis	43
Aqueous extract: VOA (30% w/w carb, 2 protein; 33.6 M% GalAc, 21.2 Ara, 18.9 Gal, 10.2 Rha, 4.7 Man, 9.4 Glu, 2 Xyl)	*Verbena officinalis* Linn.	Primary rat cortical cultures exposed to various toxins (A-beta peptides, tunicamycin, DTT, UV, and hydrogen peroxide)	25–150 µg/ml	1 hour pretreatment + 24 hours (A-beta or UV) or 16 hours (other toxins)	Caspase-2 and -3 activity; western blot (PKR and JNK phosphorylation, caspase-2 and -3); DAPI staining	50, 75, 100, and 150 µg/ml differentially attenuated cytotoxic effects of A-beta and DTT	46

Akt, serine/threonine kinase; Ara, arabinose; DAPI, 4′,6-diamidino-2-phenylindole; DCF, 2,7-dichlorofluorescin; DOPAC, 3,4-dihydroxyphenylacetic acid, DTT, dithiothreitol; ERK, extracellular signal-regulated kinase; Gal, galactose; GalAc, *N*-acetylgalactosamine, GFAP, glial fibrillary acidic protein; Glu, glucose; GluAc, *N*-acetylglucosamine; GSK, glycogen synthase kinase; HVA, homovanillic acid; Hcy, homocysteine; i.c.v., intracerebroventricular injection; ICH, intracerebral hemorrhage; i.p., intraperitoneal injection; i.v., intravenous injection; JNK, c-Jun N-terminal kinase; LDH, lactate dehydrogenase; Man, mannose; MAP-2, microtubule-associated protein 2; MAPK, mitogen-activated protein kinase; MPO, myeloperoxidase; MPP + , 1-methyl-4-phenylpyridinium; MPTP, 1-methyl-4-phenyl-1,2,3,6-tetrahydropyridine; MRI, magnetic resonance imaging; MTT, 3-(4,5-dimethylthiazol-2-yl)-2,5-diphenyltetrazolium bromide; PDK-1, 3-phosphoinositide-dependent kinase 1; PK, parkin; PKC, protein kinase C; PKR, protein kinase R; Rha, rhamnose; RT-PCR, reverse transcription polymerase chain reaction; TH, tyrosine hydroxylase; tNhtt-eGFP, truncated N-terminal huntington fused to an enhanced green fluorescent protein; UV, ultraviolet; VAchT, vesicular acetylcholine transporter; Xyl, xylose.

Effects of the disaccharide trehalose, found in baker's yeast, have been studied in a mouse model of Huntington's disease, R6/2 transgenic mice. Following oral administration for 12 weeks, trehalose improved motor dysfunction and reduced brain atrophy and the number of ubiquitin-positive aggregates in the motor cortex and striatum.^32^ A comparative glucose feeding was found to be ineffective, leading the authors to conclude that the beneficial activities of trehalose could be attributed to the trehalose molecule itself and not to its glucose subunits. Indeed, trehalose was found in measurable amounts in brain homogenates. *In vitro* treatment utilizing a cellular model for Huntington's revealed that trehalose, as well as the oligosaccharide *N*-acetylgalactosamine tetramer, the disaccharide maltitol, and the monosaccharide mannose, each decreased the amount of polyglutamine aggregates and/or improved cell survival, with trehalose proving to be the most effective.^32^ A number of additional sugar molecules showed no beneficial effects, including glucose, *N*-acetylneuraminic acid, sucrose, turanose, cellobiose, melibiose, and melezitose. In a second study, significant improvements in motor function as well as memory performance were seen in R6/2 mice when treated with a combination of orally administered trehalose along with intracranial ventricular injections of neural progenitor cells.^33^ The authors of both studies hypothesize that trehalose may be acting by directly binding to polyglutamine proteins in the brain and inhibiting aggregation. Trehalose consumption has also been shown to be beneficial in a mouse model of tauopathy with Parkinsonism by improving motor function and anxiety-related behavior and decreasing tau pathology through an apparent increase in autophagic processes.^34^

The polysaccharide fucoidan may be another candidate for neurodegenerative disease therapies. In an *in vitro* model of Parkinson's disease, fucoidan from the brown alga *Laminaria japonica* protected mouse dopaminergic MN9D cells from 1-methyl-4-phenyl-1,2,3,6-tetrahydropyridine (MPTP) toxicity. In an MPTP-exposed mouse model of Parkinson's, daily intraperitoneal injections of *Laminaria* fucoidan reduced locomotor deficits, prevented striatal depletion of dopamine, enhanced brain antioxidant activity, and protected against the loss of dopaminergic neurons in the substantia nigra.^35^ In acutely dissociated rat basal forebrain neurons from the diagonal band of Broca, treatment with fucoidan from *Fucus vesiculosus* (Bladderwrack) protected cells from A-beta-induced apoptosis.^36^ Preliminary research suggests that fucoidan may also protect against brain damage associated with ischemic stroke. Compared with control animals, rats receiving intravenous infusion of Bladderwrack fucoidan for 7 days following intracerebral hemorrhage induction showed improved motor function and memory retention in a passive avoidance test, despite an increase in hematoma size.^37^ The authors hypothesized that these effects were due to fucoidan's anti-inflammation or hemodilation activities, although these possible mechanisms of action were not investigated in this study. Bladderwrack fucoidan, when administered through intraperitoneal injection, was also able to attenuate the extent of hypoxia-ischemia-induced neural damage in the rat cortex, hippocampus, and striatum.^38^

Additional *in vitro* studies have examined possible neuroprotective effects of crude plant extracts rich in complex saccharides (Table 4). Four different polysaccharide fractions from *Nerium indicum* (Oleander) reduced A-beta peptide-induced apoptosis of primary rat cortical neurons, whereas three of the extract fractions also improved survival during serum-deprivation.^39,40^ Similar protection against A-beta toxicity occurred in cultured cortical neurons treated with various polysaccharide-rich extracts of *Lycium barbarum* (Wolfberry).^41–43^ Polysaccharide fractions from Wolfberry also reduced the amount of glutamate excitotoxicity and homocysteine-induced cell death in cortical cultures.^44,45^ Finally, an aqueous extract of *Verbena officinalis* Linn. (Verbena), containing 30% w/w carbohydrates, protected primary rat cortical neurons from the cytotoxic effects of A-beta and dithiothreitol. The verbena extract had no protective effects against tunicamycin, hydrogen peroxide, or ultraviolet-radiation, leading the authors to speculate that components of this extract may act as antioxidants and/or bind directly to putative cell surface receptors that can detect extracellular A-beta.^46^ In this collection of studies of saccharide-rich extracts, treatments appeared to improve neuronal survival by either inhibiting intracellular signaling molecules involved in cell death or activating survival pathways.

## Towards a mechanistic understanding of dietary saccharide-induced neurologic effects

Given the wide range of sugar compositions and structures of the saccharide compounds discussed in this review, common mechanisms of action that take place within the brain following oral consumption can be difficult to ascertain. Direct effects on the CNS would have to overcome the significant barriers of entry for complex carbohydrates that exist between the gastrointestinal tract and blood stream and then across the blood–brain barrier. Although such direct mechanisms cannot be ruled out, it is perhaps more likely that initial changes are occurring peripherally or within the digestive tract itself that ultimately result in cognitive outcomes. To elucidate possible mechanisms of action that allow ingested saccharides to affect brain function, one must have an understanding of how these compounds are processed in the gut.

Human enzymes capable of digesting carbohydrates are largely limited to alpha-amylases that are able to hydrolyze the glycosidic bonds found in starches and certain disaccharides such as maltose and sucrose. Glucose and other monosaccharide subunits are then released and absorbed into the bloodstream, with general consensus that the majority of these monosaccharides travel on to the liver to be used as energy. The disaccharide trehalose can also be hydrolyzed into its two glucose subunits by the enzyme trehalase expressed in cells along the brush border of the small intestine.^47^ Here, we have presented animal and *in vitro* studies suggesting trehalose may be a viable dietary candidate for treatment of neurodegenerative disorders associated with protein misfolding (tauopathies), although no relevant testing has yet been performed on human subjects. A comparative study of the effects of trehalose versus glucose in a mouse model of Huntington's disease appeared to rule out the possibility that trehalose provides protective benefits via an increase in blood glucose levels, since trehalose was found present within the brain and glucose administration did not show the same effects.^32^ Additional research is needed to completely rule out whether changes in blood glucose levels are occurring following consumption of trehalose or other naturally derived complex saccharides and whether these changes are responsible for the observed alterations in brain function. Recent noteworthy evidence from a human intervention study with the mixed polysaccharide supplement, Ambrotose^®^ complex, demonstrated beneficial effects of acute saccharide treatment on brain function, in terms of memory and cognitively demanding tasks, independent of any change in blood glucose response.^14^

When consumed, the vast majority of plant-derived, non-starch complex carbohydrates are considered indigestible by human enzymes and will either pass through the digestive tract intact or may be broken down and fermented by species of colonic bacteria. Many are known to have prebiotic effects, meaning they can stimulate the growth and/or activity of beneficial bacteria that reside in the digestive tract. For example, beneficial alterations in intestinal bacteria have been demonstrated in *in vitro* and animal studies of beta-glucan and Ambrotose^®^ complex.^48,49^ New and exciting research has begun to demonstrate how alterations in gut bacteria can affect animal behaviors, such as anxiety and stress-induced changes in learning and memory, as well as hippocampal BDNF levels.^50–53^ Therefore, it seems plausible that consumption of complex saccharides may bring about alterations in intestinal bacteria that result in cognitive benefits. Complex interactions between the digestive, immune, and nervous systems may be responsible for some of the mechanisms by which this can occur.

Many plant-derived polysaccharides have been shown to regulate the immune system as they pass through the intestinal tract.^54^ For example, various beta-glucans demonstrate immune stimulating effects,^55^ whereas saccharides such as those found in Ambrotose^®^ complex show anti-inflammatory effects within the gut.^56^ In the study of dietary pectin on endotoxin-induced social withdrawal presented in Table 2, the authors suggested that pectin consumption improved intestinal barrier function thereby reducing the entry of LPS into the blood stream and the subsequent influence of systemic inflammation on the brain.^19^ Once thought to be ‘immune’ to systemic inflammatory responses, it is now being discovered that the brain can be influenced by even low levels of peripherally circulating cytokines.^57^ Neuroinflammation can lead to cognitive dysfunction and is thought to be involved in the etiology of many neurologic disorders, including Alzheimer's, multiple sclerosis, and depression.^58^ Therefore, the effects of saccharide consumption on mood and cognitive function presented here may conceivably be attributed to immune signaling. Additional studies are needed, however, to determine whether immunomodulating effects of oral polysaccharides are in any way responsible for their influence on neurologic function.

Dietary polysaccharides may also impact brain function via the digestive tract due to the activation of parasympathetic nerve fibers, hormonal signaling, or additional brain–gut axis pathways.^59^ For example, the transport of indigestible fibers through the colon is largely controlled by the vagus nerve, which projects directly from the viscera to the brain stem and can indirectly activate limbic and cortical regions of the brain involved in regulating mood and cognition. Vagus nerve stimulation has been proven as a successful therapy for a wide variety of neurologic disorders, including epilepsy, depression, and dementia.^60^ Interestingly, activation of vagal afferents in rats has been shown to enhance LTP in the dentate gyrus^61^ and increase norepinephrine levels in the hippocampus,^62^ which was one suggested underlying mechanism for the effects of ingested alpha-glucans on hippocampal synaptic plasticity.^28,29^ Repeating some of the oral studies presented in this review on vagotomized animals or human subjects may help elucidate the involvement of the vagus nerve in dietary saccharide-induced neurologic effects.

Up to this point, no studies have focused on the neuroprotective effects of consuming fucoidan or any of the other polysaccharide-rich plant extracts discussed in Table 4. Although it seems unlikely that these systemically or centrally applied saccharides are acting in a similar manner to those administered orally, we found it pertinent to include them as part of our review. Often, neuroprotective compounds are first tested *in vitro* before going on to show benefits following oral administration. Heparin-derived, low-molecular weight glycosaminoglycans, which are mixtures of polysulfated oligosaccharides, have indeed shown neuroprotective effects both *in vitro* and in animal studies.^63^ Interestingly, some evidence exists that such compounds may pass through the blood–brain barrier when administered peripherally. Del Bigio *et al.*^37^ speculated that systemically applied fucoidan, a sulfated heteropolysaccharide, may provide protection against stroke-induced neurologic damage in part through its anti-inflammatory effects, which have been observed following fucoidan ingestion in animal studies.^54^ Future research is needed to explore whether fucoidan and other natural sulfated sugar molecules can provide neuroprotective and other cognitive benefits following oral administration.

## Conclusion

There is growing evidence that naturally derived saccharides have beneficial effects on brain function, demonstrated as both cellular and functional effects in terms of neuroprotection and improvements in cognition and mood. The purpose of this review was to consolidate studies on the brain-specific effects of these sugar compounds to help foster research in the area of natural complex carbohydrates and neurologic function. At present, an understanding of the efficacy and mechanisms of action for specific saccharides and saccharide-rich extracts is limited. The most promising human clinical research indicates that supplementation with a blend of plant polysaccharides can have beneficial effects on brain function related to cognitive performance and mood. In animals, various alpha-glucans have shown the most significant effects on behavior and synaptic plasticity, whereas trehalose shows significant promise for neuroprotective benefits. Encouragingly, the general safety profiles of many of these dietary polysaccharides for human consumption are positive, with few reported adverse side effects.^54^ Before recommendations can be made regarding their specific beneficial, preventative, or therapeutic applications, more robust well-controlled clinical trials are needed. Additional basic research is also warranted to better understand the range of effects of dietary glycans on brain function and behavior and to help elucidate the mechanisms behind which they may be occurring.
